# Phase Stabilities and Influence on Magnetic and Electrical Properties of the System (Mg_x_Mn_4‐x_)MnVO_8_

**DOI:** 10.1002/chem.202502654

**Published:** 2025-11-13

**Authors:** Jonas Fraune, Thomas Bredow, Sylvia Kunz, Björn Blaschkowski, Oliver Clemens

**Affiliations:** ^1^ University of Stuttgart Institute for Materials Science Materials Synthesis Group Heisenbergstraße 3 70569 Stuttgart Germany; ^2^ University of Bonn Mulliken Center for Theoretical Chemistry Clausius Institute for Physical and theoretical Chemistry Beringstraße 4 53177 Bonn Germany; ^3^ University of Stuttgart Institute for Inorganic Chemistry Pfaffenwaldring 55 70569 Stuttgart Germany

**Keywords:** charge order, mixed valent compounds, Mn_5_VO_8_, solid solution

## Abstract

Manganese vanandates have a wide range of interesting material properties, covering magnetic, electrical, and catalytic aspects. Among them, Mn_5_VO_8_ is a compound containing manganese in both its trivalent as well as divalent oxidation states with ordering of the different manganese species on different crystallographic sites. In this article, we show that the divalent manganese can be replaced by magnesium fully up to a composition of Mg_4_MnVO_8_. For low magnesium content, this results in a change of symmetry from triclinic to monoclinic, whereas for Mg_4_MnVO_8_, a trigonal modification can be found in addition to a monoclinic phase. Density Functional Theory based calculations reveal that for magnesium rich compositions, monoclinic and trigonal modifications are energetically similar. Magnetic characterization reveals that the materials are paramagnetic around room temperature. Further, the magnesium substitution results in a strong decrease of electrical conductivity with an increase of the activation energy determined by electrochemical impedance spectroscopy.

## Graphical abstract

Substitution of divalent manganese by magnesium in Mn_5_VO_8_ was found to introduce a change of structure and electrical conductivities.

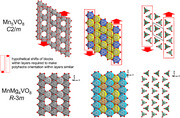

## 1 Introduction

Manganese vanadates represent an intriguing class of compounds due to their diverse electrical, magnetic, and structural properties, making them promising candidates for a variety of applications. Transition metal vanadates in general like (MO)_n_V_2_O_5_ (M = Mn, Co, Ni, Cu, Zn) are known to have potentially interesting magnetic and electrical properties.^[^ ^1^ ^]^ The copper vanadates for example can also serve as potential anode materials in the photoelectrochemical splitting of water and thus show potential for the use in oxygen electrodes for H_2_ formation.^[^ ^2^ ^]^ Manganese vanadates, especially in combination with lithium, are also of interest for applications in the field of battery materials.^[^ ^3^, ^4^ ^]^

**Figure 1:**
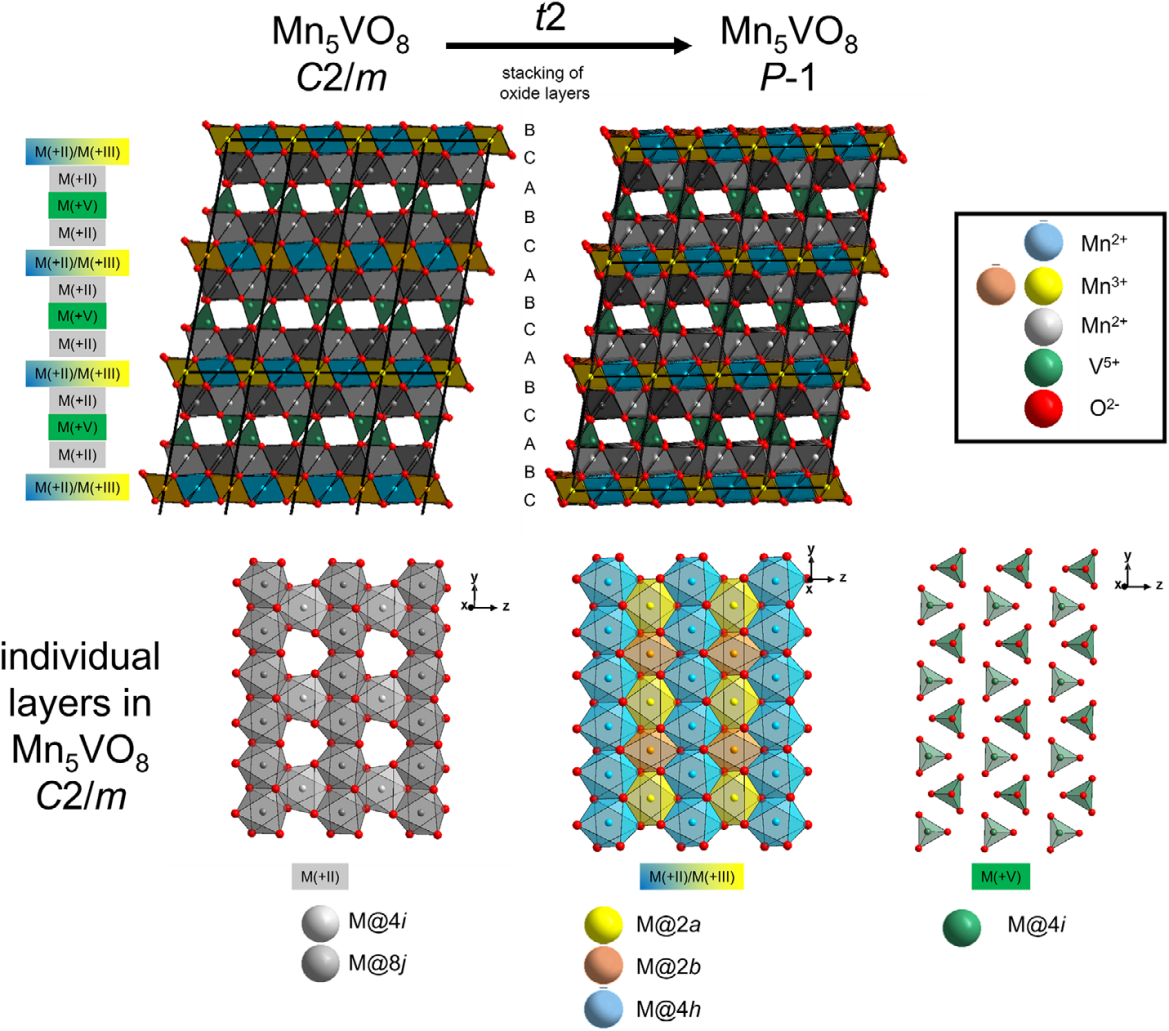
Structural relationship of monoclinic and triclinic Mn_5_VO_8_ together with coordination scheme of individual layers within the ccp stacking of oxide ions (exemplarily shown for the monoclinic modification).

In the tertiary Mn‐V‐O system, a broad range of compounds is known. These include compounds with vanadium in an oxidation state lower than + 5 combined with divalent manganese, as found in MnVO_3_^[^ ^5^ ^]^ or MnV_2_O_4_.^[^ ^6^ ^]^ Compounds with pentavalent vanadium and divalent manganese have also been reported, such as MnV_2_O_6_,^[^ ^7^ ^]^ Mn_2_V_2_O_7_^[^ ^8^ ^]^ and Mn_3_(VO_4_)_2_.^[^ ^9^, ^10^, ^11^ ^]^ One of the less studied compounds is Mn_5_VO_8_, which was discovered by Clemens et al. in 2012.^[^ ^12^ ^]^ It is the only known compound in the system MnO‐Mn_2_O_3_‐V_2_O_5_ and contains Mn in the two different oxidation states of + II and + III in a charge ordered manner on individual crystallographic sites. Depending on the synthesis route and the MnO:Mn_2_O_3_:V_2_O_5_ ratio used, the material can crystallize in a triclinic structure with space group *P*‐1 when a stoichiometric approach is used, or in a monoclinic structure with symmetry of *C*2/*m* when a non‐stoichiometric approach is applied. In the triclinic compound (see Figure 1, right), the Jahn‐Teller‐active Mn^3+^ cations occupy two different sites (1*a* and 1*b*), both showing elongation of the octahedron. The divalent manganese species are located on a total of five distinct crystallographic sites, and blocks of composition (Mn_5_O_8_)^5−^ are separated by layers containing the pentavalent vanadium cations in tetrahedral coordination. In the monoclinic compound (see Figure 1, left), the structure can be derived as a minimal supergroup of the triclinic one. The Mn^3+^ cations still occupy two distinct crystallographic sites (2*a* and 2*b*, one within a compressed and one within an elongated octahedron), while Mn^2+^ is distributed over three distinct crystallographic sites. It is also important to note that the Mn^3+^O_6_ coordination polyhedra do not share any corners, edges or faces with the vanadate V^5+^O_4_ tetrahedra, possibly due to a limited stability of Mn^3+^ against V^5+^.

Magnesium has a similar ionic radius compared to manganese^[^ ^13^ ^]^ in the divalent state (r(Mg^2+^, CN = 6) = 72 pm, r(Mn^2+^, high spin, CN = 6) = 83 pm), resulting in the fact that many manganese‐based compounds show a structural similarity to analogous magnesium‐compounds, e.g. Mg_3_(AsO_4_)_2_^[^ ^14^ ^]^ is isostructural to the tetragonal modification of Mn_3_(VO_4_)_2_.^[^ ^11^ ^]^ This motivated us to investigate the system of (Mg*_x_* Mn_4‐_ *_x_*)MnVO_8_ in greater detail in this study. We show that the divalent manganese cations can be fully substituted by Mg^2+^, with Mg^2+^ occupying only the non‐Jahn‐Teller sites in a disordered manner with only minor site preference. The exchange of Mn^2+^ by Mg^2+^ leads to a decrease in electrical conductivity, while changes in the Curie‐constant in the paramagnetic range are consistent with the maintenance of high‐spin configuration of the manganese cations. Remarkably, a new phase was identified for the targeted composition of Mg_4_MnVO_8_, which shows trigonal symmetry of *R*‐3 *m* but does not possess suitable site multiplicity for full ordering of the Mn^3+^ cations. It is isotypic to Mg_8.5_As_3_O_16_^[^ ^15^ ^]^ or Co_7.8_As_3_O_16_^[^ ^16^ ^]^ and shows structural similarity to the known modifications of Mn_5_VO_8_. DFT‐based calculations provide an insight into stability factors, which influence the formation of the different modifications.

## 2 Experimental

### 2.1 Synthesis

Mn_5_VO_8_ was synthesized by using stoichiometric amounts of Mn_3_O_4_, MnO, and V_2_O_5_. The precursors were ground with a mortar and pestle using acetone as dispersant. This process was repeated a second time for sufficient homogenization. Approximately 200 mg of the resulting powder was pressed into a pellet with a diameter of 7 mm using uniaxial force of 14 kN. This pellet was then heated under a flow of argon (99.999 % purity) at 900 °C for 35 h, followed by repeating the procedure a second time.

For the magnesium‐containing samples, different procedures had to be used to minimize formation of impurity phases. For compounds of the system (Mg*_x_* Mn_4‐_ *_x_*)MnVO_8_ with *x* = 1 and *x *= 2, the samples were prepared following the same procedure as above while replacing the according amount of MnO by MgO. The other two substituted samples with *x* = 3 and *x *= 4 were synthesized by using stoichiometric amounts of Mn_2_O_3_, MnO, MgO, and V_2_O_5_. The precursors were ground and pelletized as before, but they were heated just once at 900 °C for 35 h in a glass ampule, which was evacuated to a pressure below 0.001 mbar and sealed prior to heating.

### 2.2 X‐ray Diffraction

X‐ray diffraction measurements were performed using a Rigaku SmartLab *X*‐ray diffractometer. The diffractometer is configured in the Bragg‐Brentano geometry and utilizes Cu K_α_ radiation (i.e., comprised of two wavelengths of 1.540 596 Å for K_α1_ and 1.544 426 Å for K_α2_, with an intensity ratio of 2:1). The diffracted radiation was measured with a HyPix‐3000 detector. All samples were prepared on a sample holder with a single crystal silicon base in powdered form with a flattened surface. The analysis of the diffractograms was carried out using Rietveld analysis in the software Topas V 6.0 (Bruker) using a fundamental‐parameters approach.^[^ ^17^ ^]^ For this, the instrumental intensity distribution was determined based on a reference measurement of LaB_6_ (NIST 660a).

### 2.3 Magnetic Measurements

For the magnetic measurements a small amount of the powdered sample (between 15 – 50 mg) was enclosed in a small gelatine capsula together with wadding to fill the remaining space. This capsula was fixed in a plastic straw and then placed in a SQUID‐VSM‐magnetometer (MPM, Quantum Design, San Diego, USA). To measure the total magnetic moment as a function of temperature, the samples were zero‐field cooled to 2 K and the total magnetic moment measured in a temperature range from 2 K to 300 K and back down to 2 K with an applied field of 100 Oe. To investigate the magnetization as a function of the applied field, the samples were cooled to a temperature of 5 K. The hysteresis was then measured in a loop with the applied field ranging from 55 000 Oe to ‐55 000 Oe.

### 2.4 Impedance Measurements

For measuring the impedance spectra of the five substituted compounds, approximately 800 mg of each of the previously successfully prepared samples were pressed with a force of 20 kN to form a pellet with a diameter of 6 mm and a thickness of approximately 0.7 mm to 0.9 mm. These were then each sintered again for 15 h at 900 °C in an argon stream to obtain a dense and stable pellet. The circular surfaces of each pellet were then coated with platinum by sputtering to ensure the conductivity of the surface. The impedance was then measured using a Biologic MTZ‐35 impedance analyzer in a frequency range of 10 MHz – 100 mHz at different temperatures in steps of 25 °C ranging from 50 °C to 150 °C. Using the resistance at low temperatures, the conductivity was calculated and plotted as an Arrhenius plot to determine the activation energies for all five samples.

### 2.5 Theoretical Calculations

To complement the experiments, we performed a theoretical study at density functional theory (DFT) level. Structural and thermodynamic properties of Mn_5_VO_8_ and Mg_4_MnVO_8_ were calculated with the crystal orbital program CRYSTAL23,^[^ ^18^ ^]^ employing the hybrid functional PW1PW^[^ ^19^ ^]^ and the solid‐state triple‐zeta basis sets pob‐TZVP‐revs.^[^ ^20^ ^]^ This combination of method and basis set has proven to provide reliable results for lattice parameters of a large variety of inorganic solids.^[^ ^21^ ^]^ As recommended by the developers of CRYSTAL, the integral tolerances for the Coulomb and exchange series were set to strict values 10^−9^,10^−9^,10^−9^,10^−18^,10^−54^. Dense Monkhorst‐Pack k‐point meshes of 8 × 8 × 8 were applied. For both compounds ferromagnetic (FM) and ferrimagnetic (FIM) states were investigated. FIM spin configurations were approximated by antiparallel alignment of the atomic spins of Mn atoms occupying different Wyckoff positions. In FM states all spins of Mn atoms are aligned parallel.

## 3 Results and Discussions

### 3.1 Synthesis Results

**Figure 2:**
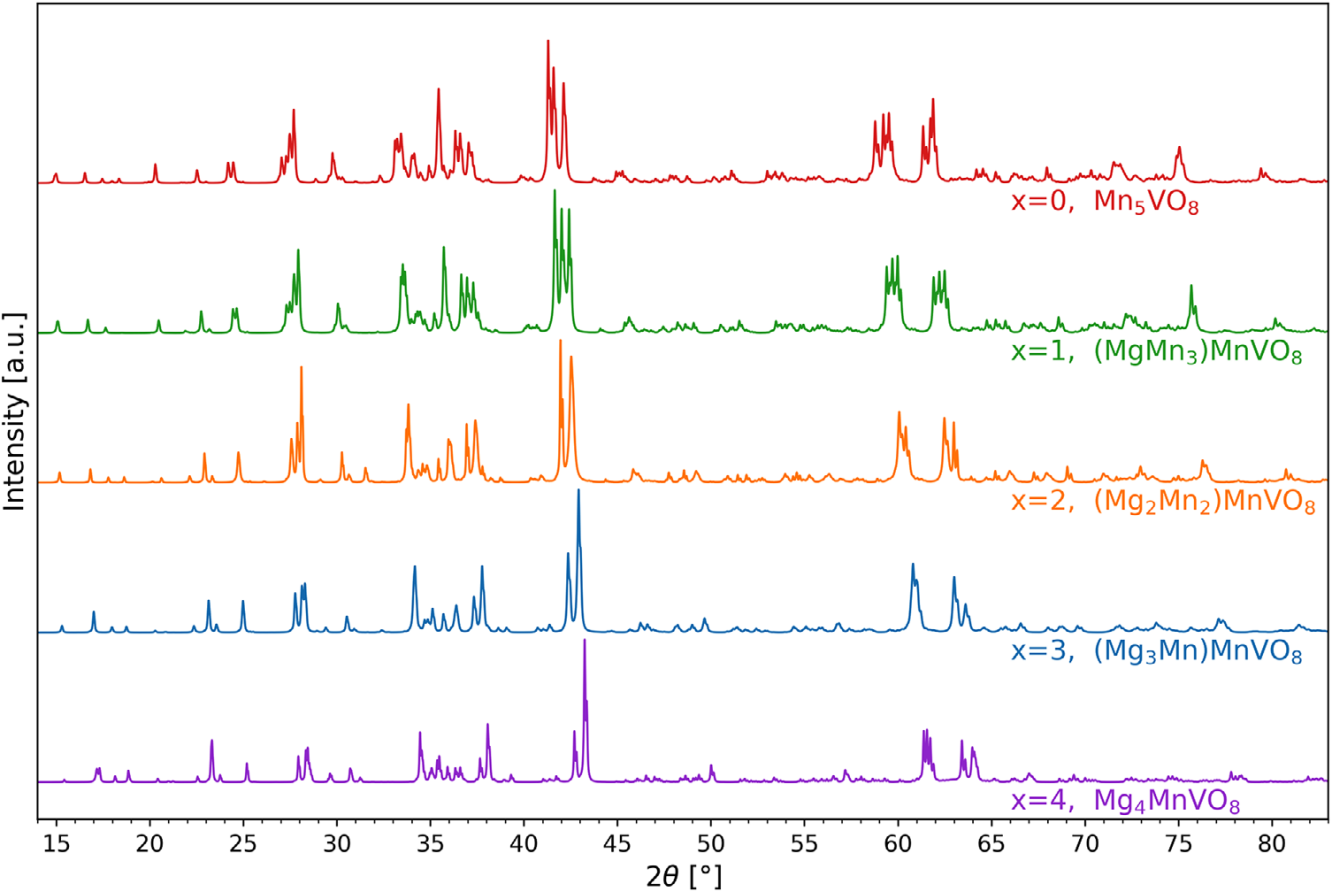
Diffraction patterns recorded for samples of composition (Mg_x_Mn_4‐x_)MnVO_8_ with x = 0–4. For better clarity, the contributions of the individual phases present can be found in the refined patterns and partial fits shown in Figure 3 and Figure S1.

**Figure 3:**
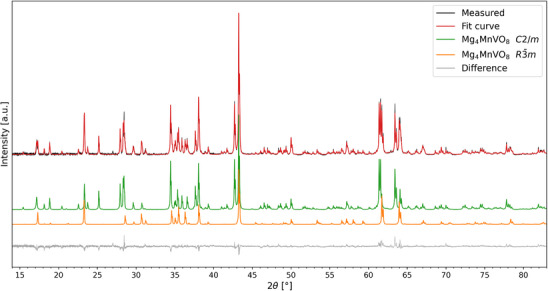
Rietveld fits of the sample with composition Mg_4_MnVO_8_ including partial fit curves showing the differences of the patterns of the monoclinic and trigonal phase present within this sample.

The XRD patterns of the five samples of (Mg_x_Mn_4‐x_)MnVO_8_ in Figure 2 show over all similarities. Groups of reflections at similar angles and with comparable intensities indicate close structural relationships. The corresponding Rietveld fits are shown in Figure S1 in the Supporting Information as well as in Figure 3.

In Table 1, the phases required to fit the complete diffraction patterns are shown along with their corresponding weight fraction and lattice parameters. In general, quantities of impurity phases that differ from (Mg_x_Mn_4‐x_)MnVO_8_ are lower than 4 wt.‐% in total for the individual samples. Mn_5_VO_8_ was received in the triclinic modification, in agreement with previous reports, and along with 2.9 wt‐% of Mn_3_O_4_. For the refinement of (MgMn_3_)MnVO_8_, two triclinic Mn_5_VO_8_‐type phases with similar lattice parameters and nearly identical cell volumes were used to fit the observed intensity pattern. This is most plausibly related to slight differences in Mn‐Mg distributions across different sites, which, however, cannot be resolved reliably for the minority phase. Both phases show a decrease in lattice parameters and cell volume per (Mg_x_Mn_4‐x_)MnVO_8_ formula unit compared to Mn_5_VO_8_. This trend continues for increasing magnesium content, showing a volume decrease of approximately 6–8 Å^3^ per Mg ion introduced per formula unit. Moreover, the angle *γ* gets closer to 90°, indicating that both phases transition toward the higher symmetry monoclinic Mn_5_VO_8_‐type structure. For *x* = 2, this transition is complete, as indicated by the apparent merging of reflections. The resulting phase can be refined using the monoclinic structure of Mn_5_VO_8_. Increasing the Mg content to x = 3 leads to a further reduction in cell volume. Again, two monoclinic phases (one majority and one minority phase) were required to refine the pattern, indicating slight inhomogeneity of Mn/Mg distribution in different grains. Since Mg and Mn have significantly different atomic form factors, we examined the possibility of site preference for the different cations. Clearly, the Mn^3+^ sites of Mn_5_VO_8_ did not show significant occupation by Mg^2+^ and were therefore constrained to be occupied only by Mn^3+^. This is plausible due to the Jahn‐Teller nature of Mn^3+^ and the corresponding requirement of typically elongated or contracted octahedra, as observed in the different modifications of Mn_5_VO_8_. For remaining sites, an occupancy scheme as shown in Table 2 was identified. In this analysis, minority phases were constrained to adopt the same composition and positional parameters as the majority phase, with deviations allowed only for the lattice parameter. This indicates only minor site preferences, with Mg^2+^ and Mn^2+^ being found on all different sites, though some sites seem to contain a lower/higher amount of Mg^2+^ than expected statistically.

**Table 1:** Appearing phases and the refined lattice parameters for the different syntheses. V_f.u._ refers to the volume per (Mg_x_Mn_4‐x_)MnVO_8_ formula unit.

*x*	Phase	Quantity [wt.‐%]	*a* [Å]	*b* [Å]	*c* [Å]	*α* [°]	*β* [°]	*γ* [°]	Volume [Å^3^]	V_f.u._ [Å^3^]
0	$P\bar{1}$	97.1	5.4319(1)	6.2058(1)	10.2430(1)	107.015(1)	99.468(1)	90.437(1)	325.11(1)	325.11
	Mn_3_O_4_	2.9	5.7628(1)	5.7628(1)	9.4619(1)	90	90	90	314.23(1)	
1	$P\bar{1}$ A	89.4	5.3851(1)	6.1635(1)	10.1688(1)	107.174(1)	99.413(1)	90.303(1)	317.61(1)	317.6
	$P\bar{1}\;$ B	10.6	5.3848(3)	6.1627(4)	10.1992(6)	107.721(4)	99.421(5)	90.153(5)	317.56(3)	317.6
2	C2/m	97.2	19.3116(1)	6.1229(1)	5.3410(1)	90	99.884(1)	90	622.16(1)	308.1
	Mn_3_(VO_4_)_2_	2.8	6.9558(1)	6.9558(1)	19.5570(1)	90	90	90	946.24(1)	
3	C2/m A	79.7	19.1691(6)	6.0645(1)	5.2785(1)	90	99.769(1)	90	604.73(1)	302.3
	C2/m B	19.4	19.1812(7)	6.0760(2)	5.2618(1)	90	99.830(3)	90	607.03(4)	303.5
	Mn_3_O_4_	0.9	5.7342(1)	5.7342(1)	9.4087(1)	90	90	90	312.38(1)	
4	C2/m	71.7	19.0728(1)	6.0214(1)	5.2328(1)	90	99.712(1)	90	592.35(1)	296.2
	$R\bar{3}$ m	28.3	6.0059(1)	6.0059(1)	28.0654(1)	90	90	120	876.70(1)	292.2

**Table 2:** Occupation of the different Mn^2+^ sites by Mg^2+^ with increasing manganese content x. The overall amount of manganese was constrained to the ratio of manganese to magnesium from the weighing of precursors.

*x*	Phase	Mn3 (*2c*)	Mn4 (*2c*)	Mn5a (*2c*)	Mn5b (*2c*)
0	$P\bar{1}$	0	0	0	0
1	$P\bar{1}$	0.269(6)	0.132(5)	0.347(10)	0.252(13)
*x*	Phase	Mn3 (*4i*)	Mn4 (*4* *h*)	Mn5 (*8j*)	
2	C2/m	0.527(4)	0.321(4)	0.576(3)	
3	C2/m	0.745(4)	0.716(3)	0.769(2)	
4	C2/m	1	1	1	

**Figure 4:**
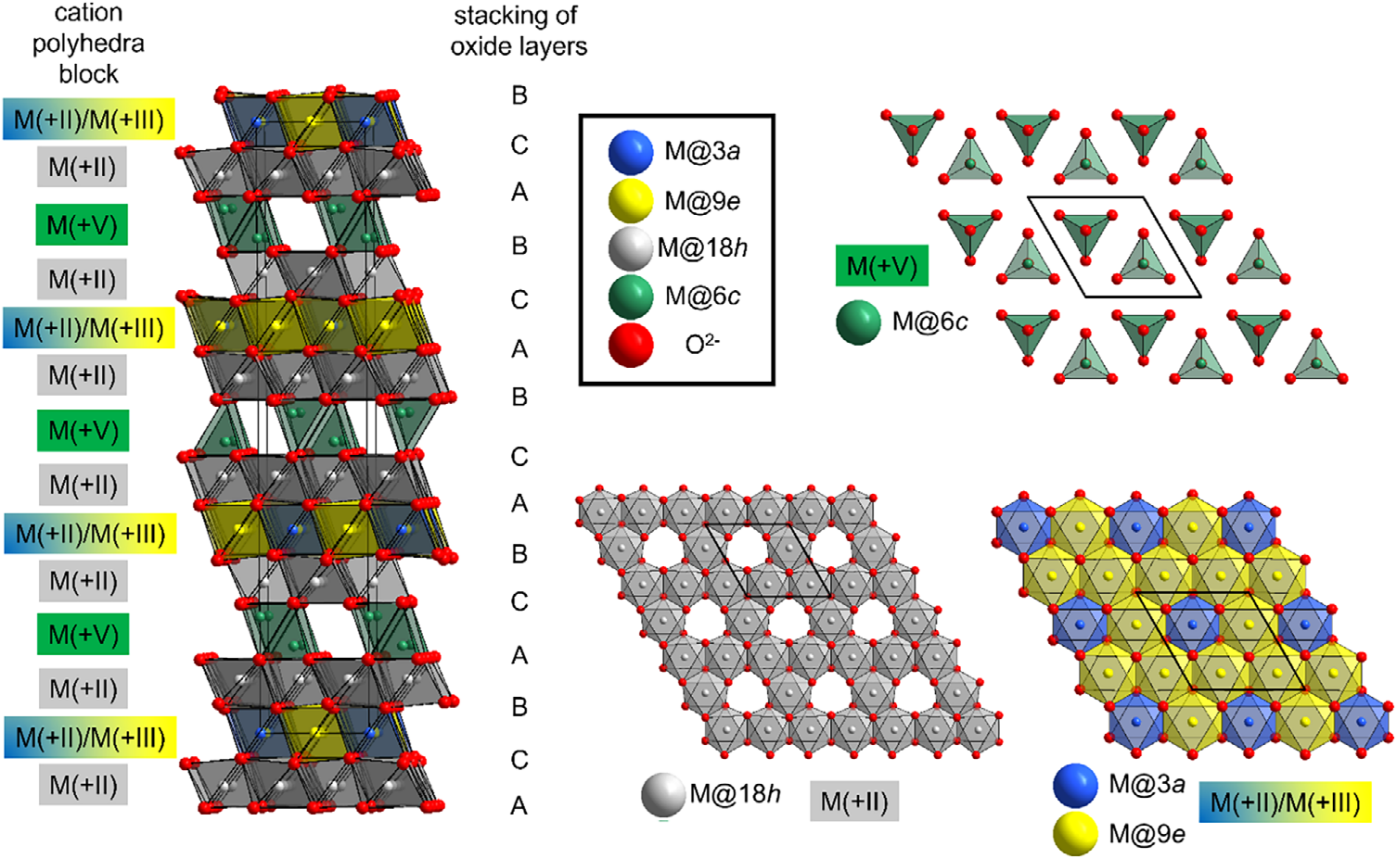
Structure of trigonal Mg_4_MnVO_8_ (R‐3 m) as determined from Rietveld analysis of powder diffraction data using a modified structural model of Mg_8.5_As_3_O_16_ as the starting model (for this compound, 6c, 3a = As, 18 h, 9e = Mg (with some vacancies within the lattice). For Mg_4_MnVO_8_, V is located at 6c, Mg is located at 18 h and partly 9e, while Mn^3+^ is located at 3a and partly 9e.

Most interestingly, the XRD pattern of the sample with x = 4 (Mg_4_MnVO_8_) indicates the appearance of an additional phase next to the monoclinic main phase (see Table 3 for the structural data), when observing the obtained difference plot of the refinement while only using a monoclinic phase. Close inspection of this difference plot, single‐peak fits of the identified sharp reflections to determine precise reflection positions, and a subsequent indexing revealed that this phase can be fitted using a Pawley fit with a trigonal *R*‐centered space group with lattice parameters of a ≈ 600 pm and c ≈ 2860 pm. Using this information, we searched the ICSD database^[^ ^22^ ^]^ for compounds with similar lattice dimensions and symmetry. In doing so, we identified a compound with the composition Mg_8.5_As_3_O_16_,^[^ ^15^ ^]^ which shows high structural similarity to Mn_5_VO_8_. It is composed of M_5_O_8_ blocks with octahedrally coordinated M‐cations (featuring a small degree of cation vacancies in Mg_8.5_As_3_O_16_), separated by tetrahedrally coordinated As^5+^ cations. Consequently, we used the structural model of Mg_8.5_As_3_O_16_, replacing the As^5+^ with V^5+^, while allowing for distribution of Mg^2+^ and Mn^3+^ on the remaining sites, which could be well used to fit the pattern in a sufficient manner (see Figure 3 for the pattern and Figure 4 and Table 4 for the structural model corresponding to this new phase).

**Figure 5:**
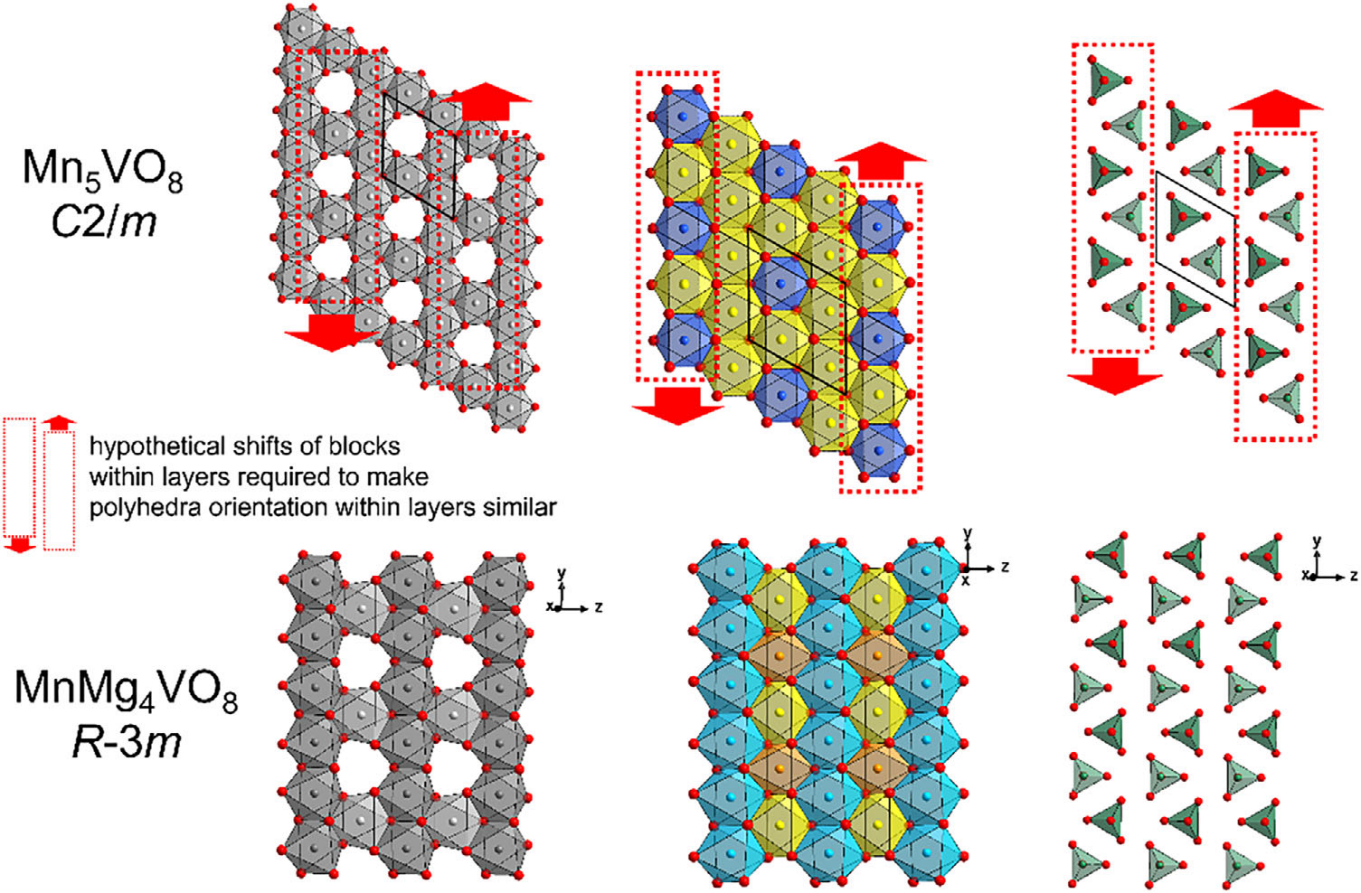
Illustration of the differences within the different layers of the monoclinic structure of Mn_5_VO_8_ and trigonal structure of Mg_4_MnVO_8_ showing similar layer composition, but different arrangement of polyhedral within layers. Red arrows and dashed boxes indicate hypothetical shifts required to transform one arrangement into the other.

Though it also contains blocks of composition M_5_O_8_^5−^ separated by V^5+^ ions, we noticed a deviating arrangement of the different sites within the individual layers resulting in a different symmetry pattern, especially visible for the tetrahedrally coordinated V^5+^ / As^5+^ species. These differences of orientation in the layers are depicted in Figure 5 and a hypothetical shift of some rows is indicated, that distinguishes the two structures. Despite their structural similarities, the translational symmetry of the monoclinc *C*2/*m*‐type and the trigonal *R*‐3*m*‐type compounds differ and do not fulfill requirements for a group‐subgroup relationship. Accordingly, the *C*2/*m* symmetry cannot be derived as a subgroup from the *R*‐3*m*‐type structure. In other words, a common supergroup of both the *R*‐3*m*‐type and *C*2/*m*‐type settings would be required to relate the structures. This common supergroup structure would be close to a rock salt scenario, i.e., near the highest symmetric aristotype structure with *Fm*‐3 *m* symmetry, which is not considered to be helpful in order to compare the structures. Further, the trigonal phase has a volume which seems to reduced by further 4 Å^3^ per formula unit than found for Mg_4_MnVO_8_, which most likely originates from the different packing density and the overall higher symmetry allowing for more close packing.

We also attempted to refine the site occupancies of the various octahedrally coordinated sites in the *R*3‐*m*‐type Mg_4_MnVO_8_. Given that this is a minority phase and has not yet been obtained in phase‐pure form, the resulting values should be interpreted with caution. Nevertheless, they provide some interesting structural insights. First, it is evident that the site multiplicities in the *R*‐3 *m* model (see Table 4 for the structural model of Mg_4_MnVO_8_ with *R*‐3*m*‐symmetry) require a mixed site occupancy of at least one crystallographic site by both Mg and Mn species. The results of the structural refinements indicate that the 3*a* site favors occupancy by the heavier scattering cation Mn^3+^, while the 18 *h* site favors the occupancy by Mg^2+^. Thus, the occupancy of the 9*e* site was constrained to ensure overall agreement with the composition Mg_4_MnVO_8_. This distribution of cations is consistent with the previous observations that Mn^3+^ and V^5+^ species tend to occupy polyhedral, which are not directly connected to each other.

**Table 3:** Structural data observed for the monoclinic compound Mg_4_MnVO_8_.

site label	atom type	Wyck. site	site symmetry	site occupancy	x	y	z
M@2a	Mn^3+^	*2a*	2/*m*	1	**0**	**0**	**0**
M@2b	Mn^3+^	*2b*	2/*m*	1	**0**	**^1^/_2_**	**0**
M@4i	Mg^2+^	*4i*	*m*	1	0.6311(1)	**0**	0.7535(6)
M@4h	Mg^2+^	*4h*	2	1	**0**	0.2449(6)	**^1^/_2_**
M@8j	Mg^2+^	*8j*	1	1	0.6318(1)	0.7449(4)	0.2527(4)
V	V^5+^	*4i*	*m*	1	0.2123(1)	**0**	0.8013(4)
O1	O^2−^	*8j*	1	1	0.1880(2)	0.7690(6)	0.6187(8)
O2	O^2−^	*8j*	1	1	0.5578(2)	0.2264(7)	0.8881(8)
O3	O^2−^	*4i*	*m*	1	0.4349(3)	**0**	0.5969(11)
O4	O^2−^	*4i*	*m*	1	0.9431(3)	**0**	0.6451(10)
O5	O^2−^	*4i*	*m*	1	0.1843(3)	**0**	0.0847(11)
O6	O^2−^	*4i*	*m*	1	0.6896(3)	**0**	0.1325(11)
***C*2/*m***, *a* = 19.0728(1) Å, *b *= 6.02134(3) Å, *c* = 5.23284(4) Å*; phase fraction 71.69 wt‐%*							
R_bragg_ 1.51 %			R_wp_ 2.57 %			GOF 1.26	

The reason for the two‐phase mixture in the sample is still unclear. One possible explanation could be a reconstructive phase transition from one modification to the other at higher temperatures, which would require rearrangement of the cations within the individual layers and might therefore be kinetically hindered. Another explanation could be that the more disordered trigonal phase forms due to kinetic factors during the synthesis process. Identifying the detailed reason for the two‐phase nature experimentally would likely require high temperature diffraction analysis, which is beyond the scope of this study. However, DFT‐based calculations reported and discussed in more detail in the following Section 3.2 indicate that the monoclinic phase has a higher thermodynamic stability even for a Mg‐rich composition.

### 3.2 DFT Calculations for Stabilities of Different Modifications of Mn_5_VO_8_ and Mg_4_MnVO_8_

In addition to the synthesis experiments, DFT calculations were performed to compare the stability of the triclinic and monoclinic phases of non‐substituted Mn_5_VO_8_. The structures experimentally determined by Clemens et al.^[^ ^12^ ^]^ were used as the starting point for the structural optimizations of the polymorphs of Mn_5_VO_8_. The calculations were performed using an open‐shell approach and various reasonable spin configurations were tested in the optimizations. For the monoclinic structure, a ferrimagnetic configuration (FIM) was found to be the most stable, in which the two Mn^3+^ ions have a different spin orientation than the Mn^2+^ ions. For the triclinic structure, however, an antiferromagnetic configuration (AFM) with alternating spins proved to be the most stable. The relative thermodynamic stability of the trigonal (*R*‐3 *m*), monoclinic (*C*2/*m*), and triclinic phases (*P*‐1) were assessed by calculating their Gibbs free energies *G_298_*. For each phase, frequency calculations of the fully optimized structures were performed, and the vibrational contributions to the thermodynamic functions *H, S, and G* were calculated by applying statistical thermodynamics and added to the electronic energy *E*. In order to reduce the computational effort, the frequency calculations were all performed for the FM states. The relative electronic energies Δ*E* of the three polymorphs are similar in the FM and FIM states. It is therefore expected that this also holds for Δ*G*. In the frequency calculation of the monoclinic phase, one imaginary frequency was obtained, indicating that this structure might not be a local minimum. The triclinic phase, however, is only slightly more stable than the monoclinic phase (Δ*G*_298_(monoclinic‐triclinic) = 1.5 kJ/mol). This difference is smaller than the expected error range of DFT methods (>5 kJ/mol). The trigonal phase is considerably less stable than the other two phases, Δ*G*_298_(trigonal‐triclinic) = 16 kJ/mol.

Similar calculations were also performed for Mg_4_MnVO_8_. In preliminary calculations, the most stable distribution of Mn over the various Wyckoff sites was identified for the trigonal phase. In agreement with the experimental XRD data shown in Table 4, the most stable Mn/Mg distribution corresponds to full occupation of the 3*a* site by Mn and a 1/3 Mn occupation of the 9*e* site. Surprisingly, a 1/6 Mn occupation of the 18 *h* site is almost isoenergetic. However, in the frequency calculations, only the 3*a*/9*e* Mn distribution was considered.

**Table 4:** Structural data observed for the trigonal compound Mg_4_MnVO_8_.

site label	atom type	Wyck. site	site symmetry	site occupancy	x	y	z
M@3a	Mn^3+^	3a	−3*m*	1	**0**	**0**	**0**
M@9e	Mg^2+^	9e	.2/*m*	^2^/_3_	**^1^/_2_**	**0**	**0**
	Mn^3+^			^1^/_3_			
M@18h	Mg^2+^	18h	.*m*	1	0.4980(3)	***1‐x***	0.2463(1)
V	V^5+^	6c	3*m*	1	**0**	**0**	0.1903(2)
O1	O^2−^	6c	3*m*	1	**0**	**0**	0.1193(4)
O2	O^2−^	6c	3*m*	1	**0**	**0**	0.3826(4)
O3	O^2−^	18h	.*m*	1	0.5147(6)	***1‐x***	0.1213(2)
O4	O^2−^	18h	.*m*	1	0.5083(6)	***1‐x***	0.3742(2)
***R*‐3 *m***, *a* = 6.0059(1) Å, *c *= 28.0652(3) Å*; phase fraction 28.31 wt‐%*							
R_bragg_ 1.00 %			R_wp_ 2.57 %			GOF 1.26	

The energy difference between AFM and FM states, Δ*E*(AFM‐FM), is less than 1 kJ/mol for all three phases of Mg_4_MnVO_8_. This is different from Mn_5_VO_8_, where Δ*E*(AFM‐FM) ≈ 24 kJ/mol for the three polymorphs. The small value of Δ*E*(AFM‐FM) is an indication that Mg_4_MnVO_8_ is paramagnetic, in agreement with the magnetic measurements in Section 3.4. The monoclinic and triclinic phases are almost isoergonic (Δ*G*_298_(monoclinic‐triclinic) = ‐ 1.6 kJ/mol), with the monoclinic phase being slightly more stable. Both structures are local minima, as indicated by the absence of imaginary frequencies. Again, the trigonal phase is considerably less stable than the other two phases, Δ*G*_298_(trigonal‐monoclinic) = 18 kJ/mol.

### 3.3 Electrical Properties

**Figure 6:**
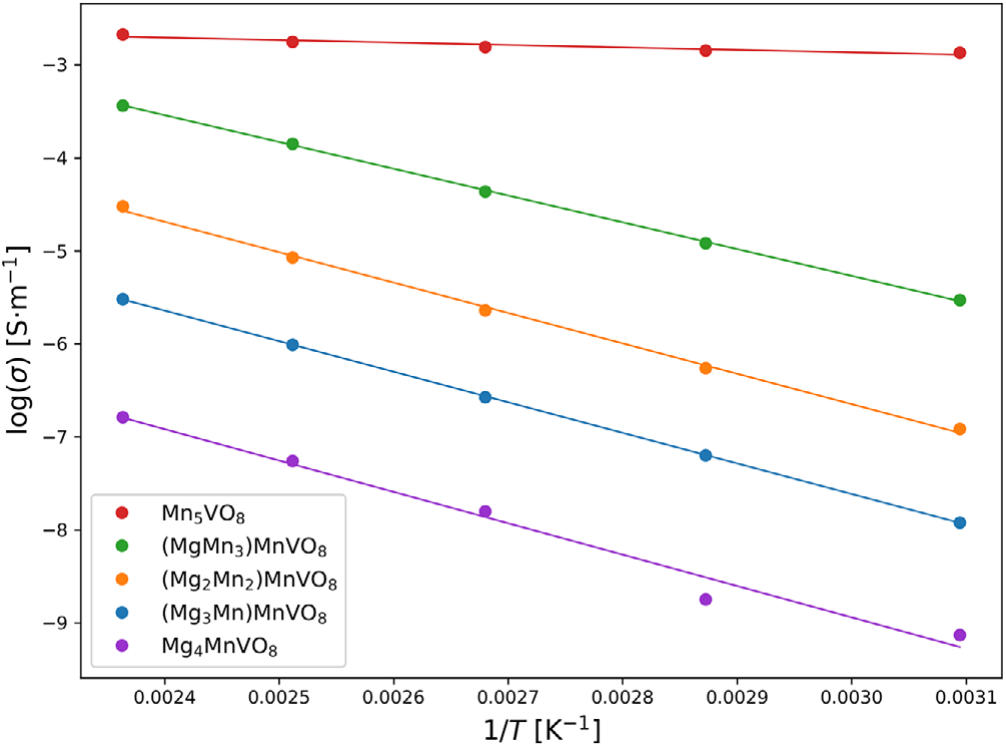
Temperature dependence of electrical conductivities of (Mg_x_Mn_4‐x_)MnVO_8_ determined from the samples’ impedances at low frequencies (Z_real_‐axis intersect).

All samples were characterized regarding their electrical conductivity by impedance spectroscopy in the temperature range between 50–150 °C (see Figure 6). For each sample a distorted semicircle can be seen in the Nyquist‐plot in Figure S2 in the Supporting Information. Although showing some unusual measurement‐dependent shapes, general information and tendencies can be drawn from them. At low frequencies, the impedance is lacking significant imaginary contributions, and no blocking response could be observed. Therefore, ohmic behavior and electronic transport can be assumed to be dominant at low frequencies. This is also visible in the Bode‐plots, where the phase difference of voltage and current response for low frequencies approaches 0° (see Figure S3 in the Supporting Information). In general, the resistance decreases drastically with increasing amount *x* of magnesium, which can be explained by the low contribution of conduction electrons by Mg. The activation energy was determined by plotting the conductivity calculated from the absolute of the impedance |Z| and sample dimensions at low frequencies against the inverse temperature in an Arrhenius plot and is shown in Table 5. The activation energy increases from 0.052 eV to 0.572 eV on replacing 25 % of Mn^2+^ with Mg^2+^ and is then slightly increasing with further magnesium amount. This could be an indication of a change in the conduction mechanism, which would require further analysis beyond the scope of this study.

**Table 5:** Activation energy from Arrhenius plot of impedance data of samples with composition (Mg_x_Mn_4‐x_)MnVO_8_.

*x*	0	1	2	3	4
Activation energy [eV]	0.052	0.572	0.649	0.652	0.669

### 3.4 Magnetic Properties

**Figure 7:**
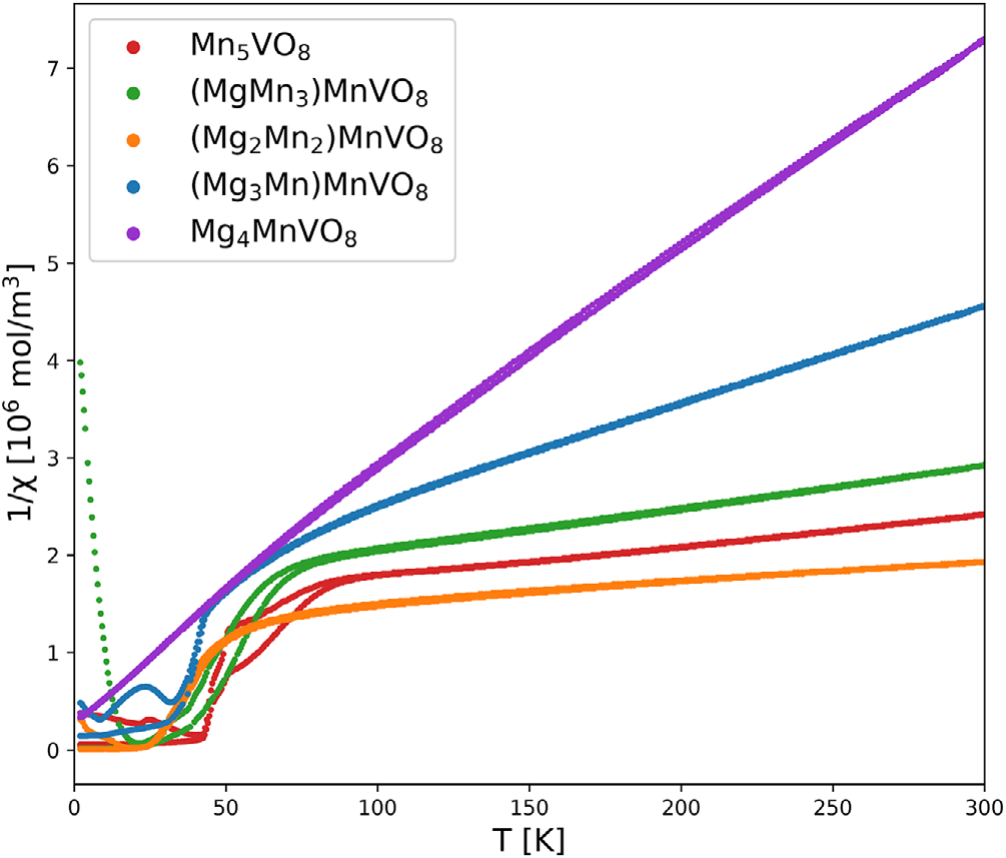
Inverse magnetic susceptibility plotted against the temperature for samples of composition (Mg_x_Mn_4‐x_)MnVO_8_.

**Figure 8:**
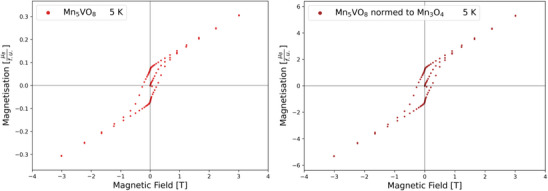
Magnetic hysteresis‐loop of Mn_5_VO_8_ (left) normed to the refined mass of Mn_3_O_4_ in the sample (right).

Magnetic measurements were performed to gain some insights into the magnetic properties and spin‐related phenomena connected to this. However, the reader must be aware that these measurements must be taken with caution, due to the presence of impurity phases and their influence on the overall measurements at low temperatures (which we will explain in the following). It was observed that all manganese‐rich phases from x = 0 ‐ 3 show a magnetic phase transition at around 50 K to the paramagnetic state, as seen in the SQUID measurements in Figure 7. In general, this transition matches the behavior of the impurity phase Mn_3_O_4_, with a Curie‐Temperature of 42.5 K^[^ ^23^ ^]^ that is present in most of the samples to a certain amount. In addition, hysteresis can be observed in magnetic field scans as seen in Figure 8, especially for the non‐substituted Mn_5_VO_8_ containing 2.9 wt‐% of Mn_3_O_4_. The zero‐field extrapolated magnetic moment of around 1.2 μ_B_ also fits that of Mn_3_O_4_,^[^ ^23^ ^]^ when normed to its refined amount. Using Curie‐Weiss‐fits in the paramagnetic region above 200 K, the magnetic moments of the samples were calculated and are listed in Table 6. They are similar to the theoretical spin‐only moments of different ratios of Mn^3+^ to Mn^2+^, which are also listed apart from the sample (Mg_2_Mn_2_)MnVO_8_. For this sample, the fitted value is too high and cannot plausibly explain a spin only moment; since this is the only composition containing the impurity phase Mn_3_(VO_4_)_2_, we assume that this might influence the determination of precise spin‐only moment from the paramagnetic regime. Thus, we conclude that for all samples the magnetic ordering temperature (if ordering takes place) would be below 50 K and would most likely be dominated by antiferromagnetic interactions, in agreement with indications given by DFT calculations. Further, magnetic transitions were absent in the fully substituted sample Mg_4_MnVO_8_, despite its dominant monoclinic structure featuring edge‐linked octahedrons, which would enable 1D magnetism in theory. Additional studies such as PPMS calorimetry in combination with SQUID measurements and/or neutron diffraction analysis might help to understand possible magnetic ordering phenomena further, but are beyond the scope of this article.

**Table 6:** Magnetic moments of the samples with theoretically expected values.

compound	Mn_5_VO_8_	(MgMn_3_)MnVO_8_	(Mg_2_Mn_2_)MnVO_8_	(Mg_3_Mn)MnVO_8_	Mg_4_MnVO_8_
μ/M	6.16	5.96	10.46	5.63	5.48
Theoretical μ/M	5.73	5.68	5.60	5.43	4.90

## 4 Conclusions

The compounds (Mg_x_Mn_4‐x_)MnVO_8_ for x = 0 ‐ 4 were successfully synthesized and structurally characterized via Rietveld analysis of powder diffraction data. Sealing the starting precursors in ampoules helped to increase phase purity in comparison to heating in a flowing Argon atmosphere. Structural variation was observed on increasing the Mg content, i.e., the structure transitions from the low symmetry triclinic modification in Mn_5_VO_8_ to a monoclinic one in (Mg_2_Mn_2_)MnVO_8_ and (Mg_3_Mn)MnVO_8_ being in agreement with DFT‐based calculations. In addition, Mg_4_MnVO_8_ was found to be close to an morphotropic phase boundary, also forming a higher symmetric, trigonal phase with different polyhedra connectivity within the individual layers partly observed in the sample; however, DFT calculations indicate that the trigonal modification is energetically less favourable compared to the monoclinic modification for Mg_4_MnVO_8_. Alongside these structural changes, a decrease in conductivity was observed on magnesium incorporation, well agreeing with the insulating behaviour of magnesium oxides; in addition, all the materials are paramagnetic at ambient conditions.

Further investigations of the materials could be devoted to address the possible 1D magnetism in monoclinic Mg_4_MnVO_8_ within the chains of edge sharing MnO_6_ octahedra, in addition to using high temperature diffraction experiments to determine the conditions for stabilizing one modification over the other synthetically. To address such structure‐property relationships, neutron diffraction measurements would be required. Further, replacing Mn^3+^ by other Jahn‐Teller active cations such as Cu^2+^ accompanied with co‐doping / co‐substitution for charge compensation might possibly lead to a series of new compounds in this rarely found structure‐type.

## Conflicts of Interest

There are no conflicts of interest to declare.

## Supplementary Material

Deposition Number(s) 2500353 (for trigonal Mg_4_MnVO_8_), 2500357 (for monoclinic Mg_4_MnVO_8_),  contain(s) the supplementary crystallographic data for this paper. These data are provided free of charge by the joint Cambridge Crystallographic Data Centre and Fachinformationszentrum Karlsruhe.

## Supporting information



Supporting Information

## Data Availability

The data that support the findings of this study are available from the corresponding author upon reasonable request.
